# Representational momentum of biological motion in full-body, point-light and single-dot displays

**DOI:** 10.1038/s41598-023-36870-2

**Published:** 2023-06-28

**Authors:** Elena Zucchini, Daniele Borzelli, Antonino Casile

**Affiliations:** 1 Center for Translational Neurophysiology of Speech and Communication (CTNSC), Istituto Italiano di Tecnologia (IIT), Ferrara, Italy; 2 Department of Biomedical and Dental Sciences and Morphofunctional Imaging, University of Messina, Messina, Italy; 3 Laboratory of Neuromotor Physiology, IRCCS Fondazione Santa Lucia, Rome, Italy

**Keywords:** Perception, Sensory processing, Human behaviour

## Abstract

Observing the actions of others triggers, in our brain, an internal and automatic simulation of its unfolding in time. Here, we investigated whether the instantaneous internal representation of an observed action is modulated by the point of view under which an action is observed and the stimulus type. To this end, we motion captured the elliptical arm movement of a human actor and used these trajectories to animate a photorealistic avatar, a point-light stimulus or a single dot rendered either from an egocentric or an allocentric point of view. Crucially, the underlying physical characteristics of the movement were the same in all conditions. In a representational momentum paradigm, we then asked subjects to report the perceived last position of an observed movement at the moment in which the stimulus was randomly stopped. In all conditions, subjects tended to misremember the last configuration of the observed stimulus as being further forward than the veridical last showed position. This misrepresentation was however significantly smaller for full-body stimuli compared to point-light and single dot displays and it was not modulated by the point of view. It was also smaller when first-person full body stimuli were compared with a stimulus consisting of a solid shape moving with the same physical motion. We interpret these findings as evidence that full-body stimuli elicit a simulation process that is closer to the instantaneous veridical configuration of the observed movements while impoverished displays (both point-light and single-dot) elicit a prediction that is further forward in time. This simulation process seems to be independent from the point of view under which the actions are observed.

## Introduction

We live in a continuously changing environment and the ability to predict its future states has high behavioral relevance. These predictions are indeed necessary to compensate for the intrinsic delays that our sensory and motor systems have in processing the incoming information and in generating appropriate behavioral responses respectively.

In the specific case of visual perception, its predictive nature was acknowledged very early, already in von Helmholtz’s classic work^1^ and later confirmed and corroborated by several experimental findings^2–5^. A remarkable example of that is representational momentum (RM^6^), which is “the tendency for observers to misremember the stopping point of an event as being further forward in the direction of movement or change”^7^.

Representational momentum has been reported for a wide variety of experimental conditions and perceptual domains (see reviews in^8–10^). It was shown to be modulated by several characteristics of the stimulus. For example, it was shown that the degree of forward mislocalization of a moving target depends on both its velocity^11–14^ and acceleration^14^. Furthermore, RM was shown to depend on the degree of perceived “friction” and “gravitational force” experienced by the moving stimulus^15–17^. Finally, for an elliptical motion, it was reported that the displacement of the target reported along the tangential and inward directions were consistent with centripetal forces (^18^, but see^19^).

An important class of dynamic stimuli that we encounter in our everyday life is other People’s and our own movements. We are a social species and thus, we constantly engage in social interactions with our conspecifics. At the same time, we also act upon and interact with objects in our environment. As is the case with objects and events in our environment, also interacting with other people and objects relies on simulating how their and our own movements respectively will unfold in time. In agreement with that, several studies have consistently found RM also for stimuli displaying human actions and movements. An early study by Thornton and Hayes reported RM when observing stimuli of crowds moving in different contexts^7^. Furthermore, recognition performances of briefly presented images of actions are higher when the images are in temporal continuity with previously presented priming movies of those actions^20^. This effect is robust to changes of the actor in the priming movies and the test images^21^ and does not occur for biomechanically impossible movements^22^. Notably, RM has been also reported when human movements were presented by means of impoverished point-light stimuli (PLS), which are stimuli that consist of dots moving with the main joints of an actor performing different actions^23^. Although the resulting displays consist only of a set of moving dots, they nonetheless convey a compelling impression of a person moving and contain enough information to perform very complex perceptual judgments (for reviews see^24,25^). In particular, a series of studies using PLS and a temporal occlusion paradigm have shown that our perception continues to internally simulate in real-time an action during an occlusion (i.e. in the absence of the corresponding visual stimulus) with a remarkable degree of precision^26–29^.

Despite considerable progress, several questions are still open concerning how we internally represent and simulate observed movements and here we used representational momentum as an experimental tool to address them.

The first important question is whether actions observed from a third- or first-person point of view share similar internal temporal representations. In our everyday life, we observe both others’ actions, from a third-person point of view (3PP), and our own actions, from a first-person perspective (1PP). We know that, in addition to visual areas^25,30,31^, action observation engages an extended network of parieto-frontal areas (Action Observation Network, AON^32–37^). Interestingly, view-tuned responses have been reported in several areas within this network^38–43^ and several behavioral studies have revealed differences in how our brain processes action stimuli observed from different points of view^44–46^. It is thus conceivable that, actions observed from the first- and third- person view might engage internal simulation mechanisms having different characteristics.

The second important question is to understand how much the internal simulation of observed actions is driven by the characteristics of the motion per se or whether the display or perception of a moving body is necessary. Human movements are governed by a relatively small set of kinematic laws^47–50^. Behavioral studies have consistently shown that human perception is biased toward abstract motion stimuli complying with these laws, even when they contain no bodily shape^51–55^. Furthermore, brain imaging results suggest that observation of these disembodied motion stimuli also produces patterns of neuronal activity that strongly overlap with those produced by action perception^56^. One might thus speculate that representational momentum reported for human movements might be predominantly due to the kinematic characteristics of human movements and it might not thus require the explicit perception of a human body to be elicited. If, on the other hand, perception of human movements relies on the integration of both form and motion features, as suggested by an influential theoretical account^57^, then we might expect to observe differences in the RM elicited by normal and impoverished displays.

Addressing these questions poses non-trivial technical problems, as one should be able to show participants exactly the same human movements at different levels of impoverishment and from different points of view. Here, we used modern computer graphics to overcome these problems. Specifically, we motion captured a person during the performance of elliptical trajectories with his right arm. We then used these kinematic data to animate a photorealistic human avatar. We finally rendered the animations from the frontal and first-person perspectives and at different levels of impoverishment, ranging from full-body to point-light and single-dot stimuli. In this manner, we can dissociate the effects on perception of stimuli containing both form and motion features of human movements (full-body), stimuli that contain motion features only and elicit the percept of form features (point-light stimuli) and stimuli that contain only motion features of human movements (single-dot). Notably, all stimuli represented the same underlying physical motion and differed only for the point of view and visual appearance. We then embedded these stimuli into a RM experimental paradigm to address the two questions above.

## Methods

### Subjects

We performed two Experiments. Twenty-two participants (13 female, age range 22–33 years) and 15 participants (4 female, age range 21–43) participated in Experiment 1 and 2 respectively. All subjects were naïve to the purpose of the experiment, they had normal or corrected-to-normal vision and were compensated for their participation. All procedures were approved by the local Ethics Committees (Comitato Unico Provincia di Ferrara for Experiment 1 and Comitato Etico IRCCS Sicilia Sezione Centro Neurolesi “Bonino-Pulejo” for Experiment 2) and were in accordance with the guidelines of the Declaration of Helsinki. Prior to the experiment, all participants signed an informed consent form.

### Stimuli

Stimuli consisted of video clips showing a photorealistic human avatar, point-light stimuli or a moving solid shape. The animations were generated as in^58,59^. In brief, the movements of a human actor were recorded using a Vicon 612 motion capture system (Vicon Motion Systems Ltd., Oxford, UK) equipped with 9 cameras. The temporal sampling rate was 100 Hz, and the spatial error was less than 1 mm. The recorded movement was a smooth ellipsoidal motion of the right arm along the horizontal plane at a rate of approximately 1 turn per second. The recorded trajectories were low-pass filtered at 5 Hz. A 3 s interval was cropped from the data and imported into commercial software (3D Studio Max and MotionBuilder, both from Autodesk) to animate a commercial avatar model. Animations were then rendered in AVI format at 60 frames per second and at a resolution of 800 × 600 pixels.

**Figure 1:**
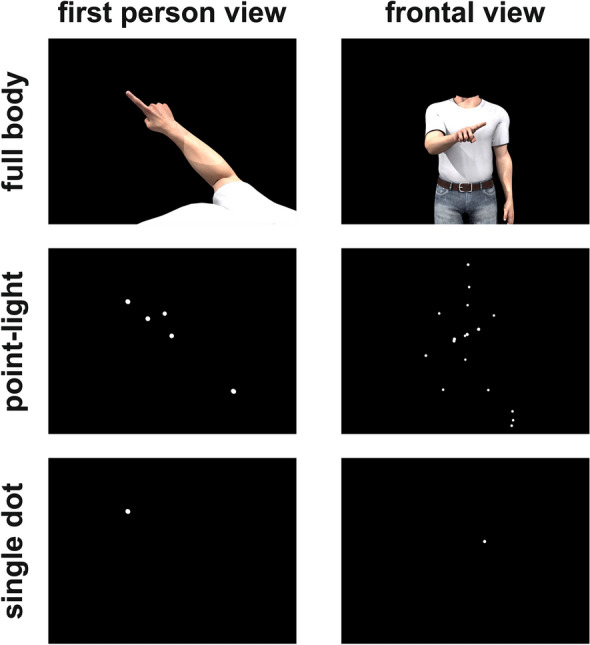
Stimuli—the six panels represent the stimuli used in the six experimental conditions respectively. They displayed a person moving his right arm along an elliptical trajectory observed from either a first-person (left column) or a frontal (right column) view. The movement was rendered either by means of a photorealistic avatar (first row), a point-light stimulus (second row) and a single dot placed on the tip of the index finger (bottom row).

We rendered a total of 7 animations showing the movements of an avatar, a point-light stimulus or a single dot as seen from a frontal or first-person point of view (Fig. 1) and the movement of a solid shape (bottom panel of Fig. 5A). The point-light stimulus was generated by parenting, in 3D Studio Max, white spheres to the major joints of the avatar, hiding the avatar body and then rendering the animation from a frontal or first-person of view (middle row in Fig. 1). Similarly, the single-dot stimulus was generated by parenting a white sphere on the tip of the index finger, hiding the avatar body and then rendering the animation of the dot from a first-person or frontal of view (bottom row in Fig. 1). The solid shape stimulus was generated by parenting a “capsule” object to the avatar forearm, hiding the avatar body and then rendering the animation of the moving object from a first-person point of view. Crucially, the physical movements of the joints, dots or solid shape was the same across all animations. The only features that changed across animations were the point of view (first-person or frontal) or the type of stimulus (avatar, point-light display, single dot or solid shape).

### Experimental paradigm

**Figure 2:**
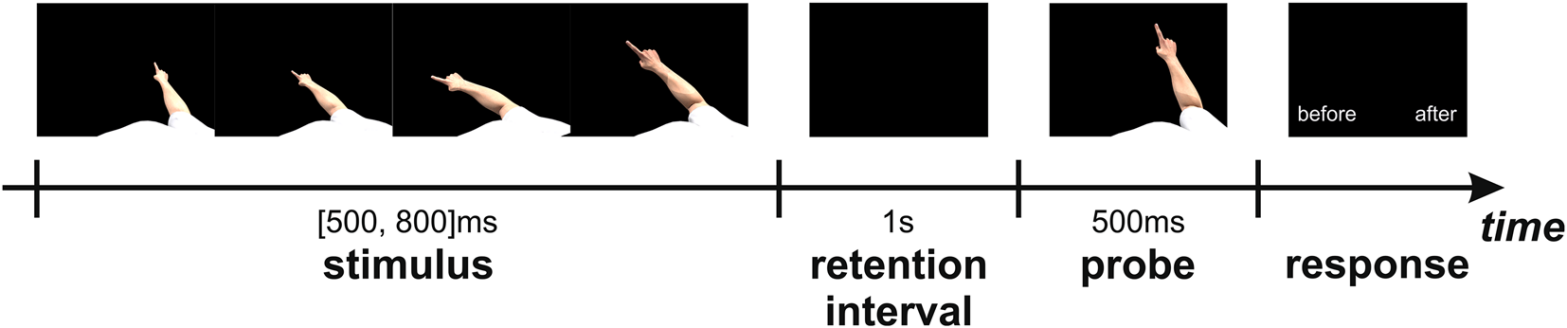
Experimental paradigm—on each trial participants were first presented with a movie representing one of the six conditions shown in Fig. 1. After a random time, uniformly distributed in the interval [500, 800] ms, the movie stopped and a blank screen was shown for 1 s. After this interval, a static frame (probe), taken from the same animation, was presented for 500 ms. Then the probe disappeared and a message appeared on the screen to ask participants to press one of two possible buttons to report whether her/his subjective perception was that the arm position in the probe followed or preceded the perceived position of the arm at the moment when the movie stopped playing. Participants received no feedback regarding the correctness of their responses.

The experimental paradigm is shown in Fig. 2. It was implemented in Octave^60^, running under Linux, using the Psychophysics Toolbox^61–63^. During the experiment, the subjects sat comfortably in front of a 21.5″ commercial LED monitor (Dell U2212HMc, 1920 × 1080 at 60 Hz) at a distance of approximately 60 cm (Experiment 1) or in front of a 17″ LED monitor (Dell Precision M6400) at a distance of approximately 50 cm (Experiment 2). Each trial began with the presentation of one of the six animations. After a random number of frames, uniformly distributed in the interval [50, 80] frames (i.e. [830, 1330] ms), stimulus presentation was interrupted and a black screen was presented for 1 s. After this time interval, a static frame was presented for 1 s. The static frame could have an offset of either − 4, − 2, 0, 2, 4 or 7 frames with respect to the last presented frame of the stimulus, where 0 indicate that it was the same frame, negative numbers indicate that it preceded it in the frame sequence and positive numbers indicate that it followed it in the frame sequence. After 1 s, the static frame disappeared and a message appeared to prompt the subjects to press the key ‘z’ or ‘m’ to indicate whether they perceived the static frame to precede or follow respectively the last presented frame of the animation. The stimulus was centered on the screen along the horizontal axis and it was shifted 50 pixels down from the center along the vertical axis. We used a asymmetric range of temporal offsets (i.e. [− 4, 7]) because, based on previous results in the field (i.e.^7,11,17^) and prior piloting studies from our lab, we expected a forward shift in time in the subjects’ responses. We thus sampled a larger range of forward displacements to allow for a better fitting of the psychometric function.

Before the experimental session, the subjects were first familiarized with the animations and explained the experimental paradigm. They were then presented with example trials by the experimenter and asked to report their percept verbally. In the example trials, the offset of the static frame was either − 10 or + 10 frames and the experimenter provided verbal feedback. A short practice session lasting few minutes followed. During this session, the offset of the static frame could be either − 5 or + 5 frames and no feedback was provided to the subjects. The practice session could be repeated at the subject’s request and it was followed by the experimental session.

In Experiment 1, during an experimental session, each combination of stimulus type (3 levels: full-body, point-light stimulus and single dot), point of view (2 levels: subjective and frontal view respectively), and offset of the static probe frame (6 levels: − 4, − 2, 0, 2, 4 or 7 frames) was repeated for 15 times for a total of 540 trials. Each subject completed two experimental sessions on different days to collect a total of 30 trials for each combination of stimulus type, point of view and offset of the probe stimulus. Experiment 2 followed the same design, with the only difference that only two stimuli (full-body and solid shape stimuli) and one point of view (first-person) were presented and, given the reduced number of experimental conditions, it was run in a single session. In both experiments, the order of conditions was randomized across subjects (Experiment 1 and 2) and sessions (Experiment 1).

### Data analysis

**Figure 3:**
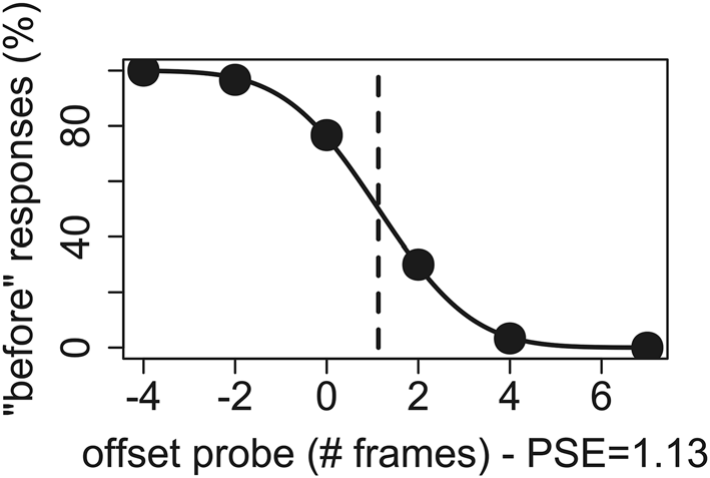
Computation of the PSE—the dots in the figure signify, for a single subject and condition, the percentages of times in which the static probe was perceived as preceding the last displayed frame of the dynamic stimulus as a function of its veridical offset (horizontal axis). The continuous line represents the fitted psychometric function and the vertical dashed line represents the point of subjective equality (PSE). That is, the offset at which the psychometric function crosses the 50% level, that, in this specific case, was 1.13.

All data analyses were performed in R^64^. For each subject, we first computed the proportion of ‘before’ responses for each combination of stimulus type, point of view and offset of the static probe frame. The dots in Fig. 3 show the results of this analysis for one of our subjects. For each combination of stimulus type and point of view, we then fitted these results with a psychometric function (see solid curve in Fig. 3) and computed the point of subjective equality (PSE), which is defined as the point where the value of the psychometric function is 0.5 (vertical dotted lines in Fig. 3). The PSE defines the value of the independent variable, which in our experiments was the offset between the static probe frame and the last presented frame, at which the subjects’ perception is perfectly unbiased between the two alternative forced choices. In representational momentum studies, it is used to index the amount of subjects’ perceptual shift^65–67^. Estimation of the PSE was performed by means of the MixedPsy R package^68,69^ using a generalized linear mixed model and a probit link function. Two participants exhibited patterns of responses that could not be fitted by a psychometric function and were thus excluded from further analysis.

In Experiment 1, we entered the PSEs computed for each subject and condition into a repeated-measures ANOVA, followed, where appropriate, by paired t-tests to further investigate effects of interest. Results of the t-tests were Holm-corrected^70^ for multiple comparisons. In Experiment 2, since only two conditions were present, we compared the PSEs by means of a paired t-test.

## Results

**Figure 4:**
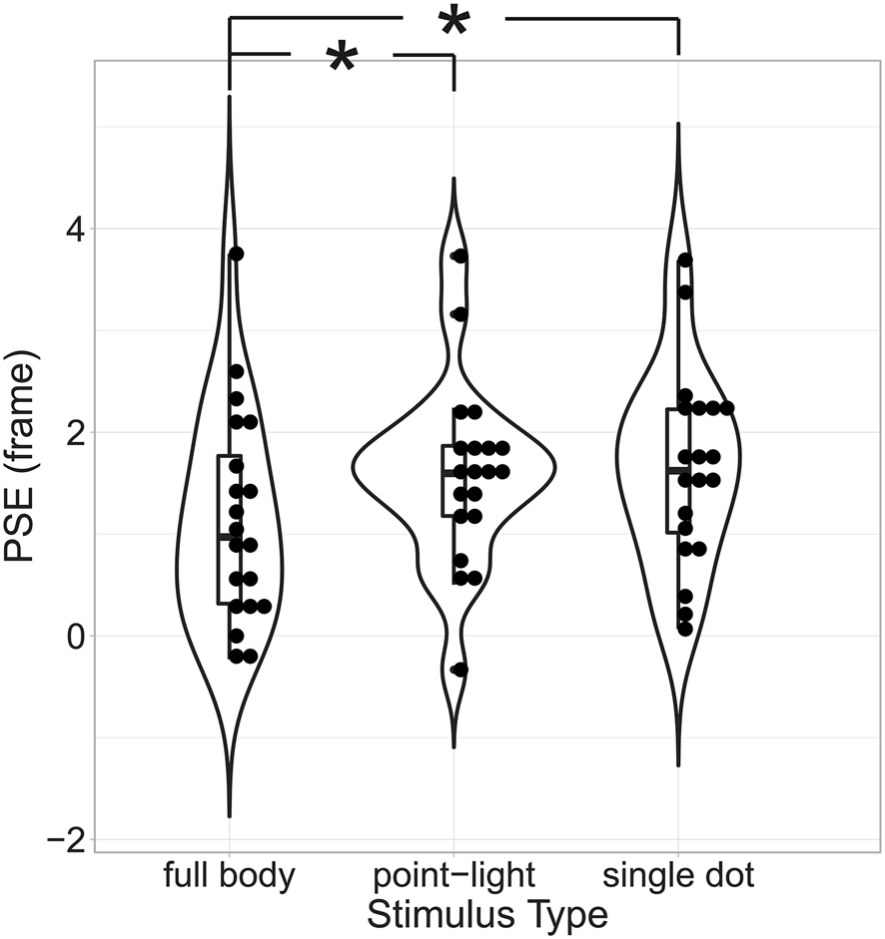
Experiment 1: RM produced by full-body, point-light and single dot stimuli—each dot in the figure signifies the point of subjective equality (PSE) estimated for a single subject and condition. The three violin plots represent the distributions of the participants’ PSEs when observing full-body (distribution on the left), point-light (distribution in the middle) and single-dot (distribution on the right) displays of human actions. We collapsed results across the factor point of view (first person or frontal) as an ANOVA analysis revealed that this factor neither produced a main effect nor was part of an interaction.

In Experiment 1, we compared PSEs computed across all subjects by means of a two-level repeated-measures ANOVA with factors point of view (2 levels: first-person and frontal view) and stimulus type (3 levels: full-body, point-light and single-dot). Results revealed a significant main effect of the factor stimulus type (F(2,38) = 4.52, *p* = 0.017), no effect of the factor point of view (F(1, 19) = 0.529, *p* = 0.48) and no interaction (F(2,38) = 1.76, *p* = 0.19). To further explore the main effect of the factor point of view (POV), we collapsed behavioral results across the factor point of view (Fig. 4. Results for all factors separately are shown in Fig. S1) and performed paired t-tests between the 3 levels of the factor stimulus type. This analysis revealed a significant difference between the conditions full-body and point-light (*p* = 0.048, corrected for multiple comparisons) and between the conditions full-body and single-dot (*p* = 0.037, corrected for multiple comparisons). No significant difference was observed between the conditions point-light and single dot (*p* = 0.759, corrected for multiple comparisons). This pattern of results suggests that the amount of RM produced by full-body stimuli is significantly smaller for full-body compared to point-light and single-dot displays. All conditions produced a positive RM and this perceptual forward time shift seems not to be modulated by whether the stimulus is observed from an egocentric or allocentric point of view.

**Figure 5:**
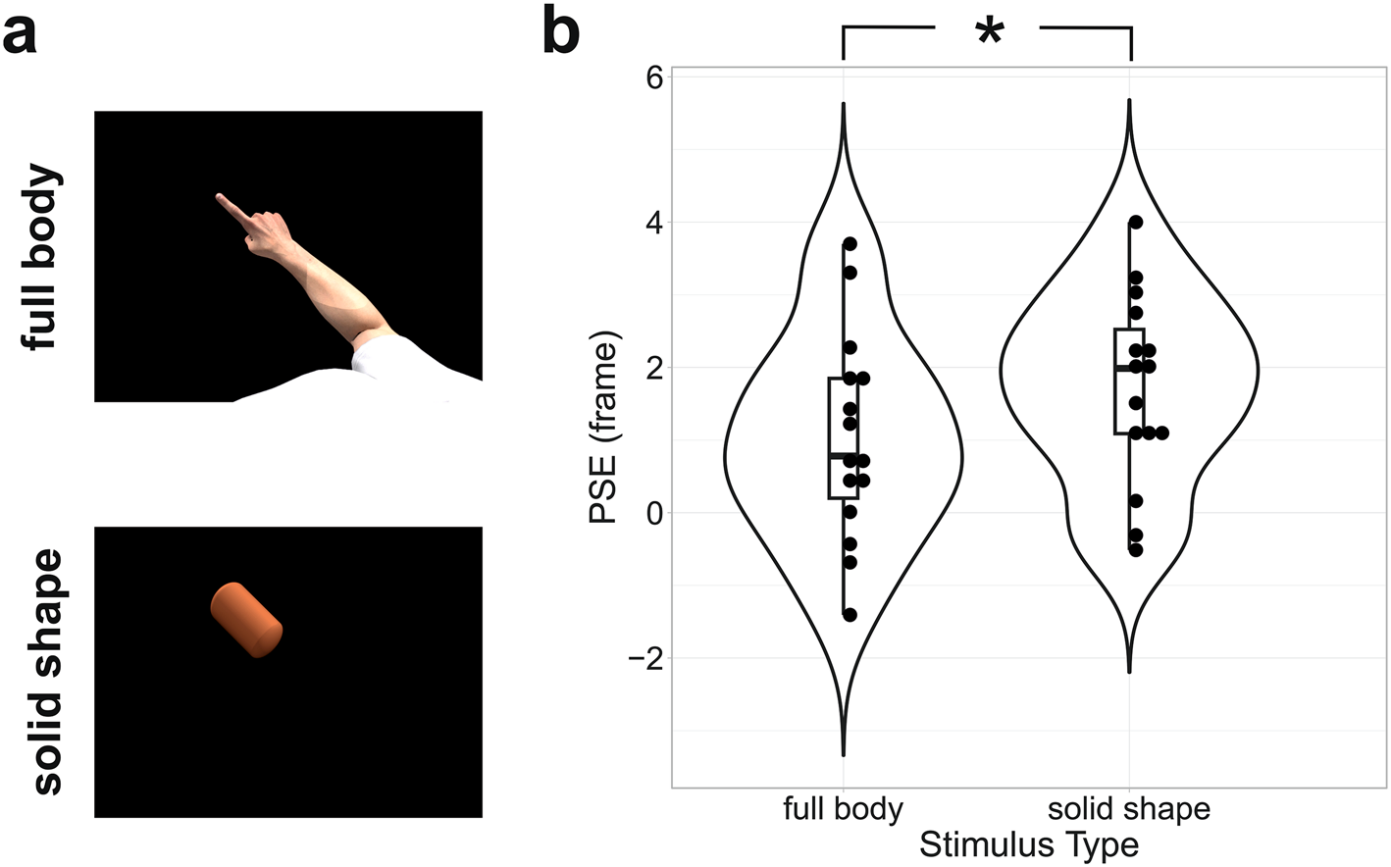
Experiment 2: RM produced by full-body and solid object stimuli—symbols and conventions are as in Fig. 4.

One possible explanation for the results in Fig. 4 is that they are predominantly due to the “richness” of the stimulus. In particular, full-body stimuli are perceptually more “salient” as they contain both form and motion information. Explicit form information is instead completely absent in both point-light and single-dot stimuli where it can only be indirectly inferred from the motion of the dots^57^. It might thus be argued that the additional low-level cues present in full-body stimuli allowed for a more veridical estimation and/or memorization of the last perceived stimulus configuration before disappearance and/or its better comparison with the probe image. To control for this possibility, in Experiment 2, we compared RM produced by full-body stimuli to that of a solid shape having the same motion and orientation of the avatar’s forearm (Fig. 5A, bottom panel). This stimulus possesses relevant low-level cues present in the full-body condition (i.e. it is “solid” and its color is determined by physically veridical illumination), while not resembling a biological effector. Given the non-significance of the factor point of view in Experiment 1, in Experiment 2 we presented only first-person stimuli. Results in Fig. 5 show that a moving solid shape produced significantly higher RM than full-body stimuli (t(14) = − 2.8, *p* = 0.014). This result suggests that the shorter RM observed for full-body stimuli cannot be explained by the richness of the visual stimulus, per se. Indeed, even the significantly visually richer solid-object stimulus still produced higher RM compared to full-body displays.

## Discussion

In this study we used representational momentum (RM^6^) to investigate the amount of forward shift in time of human perception when observing human bodily movements. In particular, we explored how this shift is modulated by two factors: the type of stimulus (full-body, point-light and single dot) in which the observed movement is embedded and the point of view (first-person or frontal view) from which it is observed. Crucially, we used high-quality computer-generated stimuli to ensure that the same underlying physical motion was presented in all conditions. The main result of our study is that observation of human bodily movements under naturalistic conditions (i.e., a full-body photorealistic avatar) seems to produce a significantly smaller forward shift in time of the observer’s perception compared to when the same physical motion is observed in impoverished displays (point-light stimulus or single-dot display). We found no effect of the point of view. These results provide new insights into how our brain perceives human movements.

The first insight is that the degree of RM elicited by human kinematics depends on the stimulus in which it is embedded. Specifically, full-body stimuli elicited a smaller, but still significantly positive, RM compared to point-light and single-dot stimuli having the same motion characteristics. The subjects’ task was to hold in memory the last perceived stimulus configuration. Thus, a smaller RM for full-body stimuli means that, for this type of stimulus, the memorized configuration was closer to the veridical one at the moment when the movie stimulus was stopped. It is important to emphasize that point-light displays, although not explicitly depicting a human body, elicit a robust and vivid perception of a moving person^23,71–75^. Thus, our results seem to suggest a “gradient” in how precisely we internally simulate observed actions, with full-body stimuli being more “veridically” simulated than point-light displays. Our result of no difference between point-light and single-dot displays seem to suggest that the former, although also producing a vivid perception of a moving body, engages prediction mechanisms that are more similar to those that would be engaged by a “disembodied” (i.e., containing or eliciting no perception of a moving body) motion.

As mentioned in the Introduction section, observation of full-body and point-light stimuli produces different response patterns at the neuronal level. That is, full-body stimuli elicit robust responses both in temporo-occipital visual areas and in parieto-frontal visuo-motor areas of the action observation network (AON; see for example^76^). On the contrary, point-light displays seem to engage mostly visual and cerebellar areas^25,57,77–81^, with only few studies revealing activations within the AON^79,82^. In previous studies, we showed that while both full-body and abstract dots displays complying with normal human kinematics produce activation within the AON^56,58^ only for full-body displays these activation patterns are time-locked to the velocity profile of the movement^59^. Consideration of these results might provide an explanation for the smaller RM produced by full-body compared to single-dot displays reported here. Indeed, we can hypothesize that, in our experiments, the widespread and time-locked activation produced within the AON by the full-body stimuli, when compared to the point-light and single-dot displays, allowed a temporally more veridical internal simulation of the observed action. This more precise internal simulation might be the reason behind the observation of a more accurate estimation of the stimulus configuration at the time when the movie stimulus was stopped.

The second insight provided by our study, is that our internal simulation process does not seem to be tuned for the point of view. Indeed, in our experiments, the factor point of view neither produced a main effect nor was involved in any interaction. In particular, we found that both the first and the third person point of view produced a forward temporal shift in judging the state of the stimulus at the time of its disappearance. This pattern of result is in accordance with a RM study by Brattan et al.^67^. In their experiments, Brattan et al. asked subjects to judge the correct continuation of a human action after an occlusion. In agreement with our results, they found a positive forward prediction bias, both for actions presented from the first- and the third-person points of view. This bias was not statistically different between the two perspectives. Our study extends this result to point-light and single-dot stimuli, thus corroborating the hypothesis that actions observed from different points of view produce internal simulation processes with similar temporal dynamics. Our results do not however imply that this simulation process is a-modal. There is, on the contrary, experimental evidence suggesting that the point of view is an integral component of how we internally represent and simulate actions. For example, Jarraya et al. found that viewpoint changes interfered with the detection of motion discontinuity in point-light displays of human movements. In a similar vein, Campanella et al.^66^ found that the discrimination of the size of an unseen object from the observed hand configuration was better in egocentric compared to allocentric view^44^. Finally, previous neurophysiological studies from our lab, showed that a large percentages of mirror neurons^33,37,83–85^, one of the putative neuronal substrates for the simulation of others’ actions, exhibit view-tuned responses^38,39^. Taken together, these results and the present study suggest that while several aspects of how we internally *represent* observed actions are view-dependent, how we internally *simulate* their unfolding in time appears instead to be view-independent.

The third insight concerns the amount of forward shift in time of the instantaneous internal representation of an internal action. In our experiments, we found that this shift was, on average, 1.15 and 1.03 frames in Experiment 1 and 2 respectively for full-body stimuli, 1.59, 1.64 and 1.71 frames for point-light, single-dot and solid shape displays, respectively. These values correspond to 17 ms and 19 ms (full-body, Experiment 1 and 2 respectively), 26 ms (point-light), 27 ms (single-dot) and 29 ms (solid shape) at the rate of 60 frames per second that we used in our experiments. They are in line with previous experiments^67,86^ and they further suggest that our perception of observed actions has a remarkable degree of temporal precision.

A potential additional explanation for the lower RM in the full-body condition could be that the richer content of the visual stimulus in this condition, containing both form and motion features, provided more information to the perceptual comparison of the memorized last frame of the movie and the probe stimulus. Results of Experiment 2, showing that RM produced by a solid object was significantly greater than that produced by full-body stimuli, suggest that the “richness” of the stimulus, per se, cannot explain our results. Of course, our control experiment could not necessarily investigate all potential visual features that might modulate RM and future experiments are needed to fully explore this point.

It is worth emphasizing that, to our best knowledge, this is the first study that quantitatively compares RM elicited by full-body, point-light and single-dot stimuli displaying the same underlying physical motion and using the same experimental paradigm. Previous studies used either full-body or point-light stimuli and different experimental paradigms (e.g.^67,87^). These methodological differences prevented a direct comparison of experimental results. Here, we used instead the same paradigm for all stimuli types and we were thus able to directly compare RM across conditions. This comparison generated several key insights as discussed above. Notably, our approach to leverage modern computer graphics to render the same human movements at different levels of impoverishment can be used to probe additional characteristics of RM during the observation of human movements. For example, one interesting next step will be to investigate whether RM of full-body and impoverished stimuli is modulated by compliance or not of the stimuli with normal human kinematics. From previous studies we know that displays of human movements and abstract motion stimuli activates different brain networks depending on whether the stimuli comply or not with kinematic invariants of human movements^56,58^. It would thus be interesting to test whether this difference at the neuronal level translates to a difference in RM at the behavioral level.

In conclusion, our results provide evidence that our perceptual mechanisms for the internal simulation of observed actions seem to be differentially engaged by different types of stimulus displays. When the stimulus is a full-body display, our instantaneous internal representation of the stimulus configuration is significantly closer to its veridical physical state than when the stimulus is a point-light, single-dot or solid shape display. Furthermore, our internal simulation mechanisms do not seem to be modulated by the point of view under which the action is observed.

## Supplementary Information

Supplementary Figure S1.

## Data Availability

Upon publication, the corresponding author (AC) will make the data collected in this study available on an open database. He will also send them directly upon request.
