# Supplementary material for: Representational momentum of biological motion in full-body, point-light and single-dot displays
Source: Sci Rep. 2023 Jun 28;13:10488. doi: 10.1038/s41598-023-36870-2 (PMC10307891; doi:10.1038/s41598-023-36870-2)
Supplement: Supplementary file 1 — Supplementary Figure S1. [file 41598_2023_36870_MOESM1_ESM.pdf]

Supplementary material

Representational momentum of biological motion in full-body, point-light and single-dot displays

Elena Zucchini[*,2,3], Daniele Borzelli[*,1,4] and Antonino Casile[1,2,3,5]

[1] University of Messina,
Department of Biomedical and Dental Sciences and
Morphofunctional Imaging,
Messina, Italy
[2] Istituto Italiano di Tecnologia, (IIT)
Center for Translational Neurophysiology of Speech and Communication (CTNSC)
Ferrara, Italy
[3] Section of Physiology
Department of Neuroscience and Rehabilitation
University of Ferrara,
Ferrara, Italy
[4] IRCCS Fondazione Santa Lucia,
Laboratory of Neuromotor Physiology,
Rome, Italy

[*] Equal contribution

[5] Corresponding author: toninocasile@gmail.com, antonino.casile@unime.it

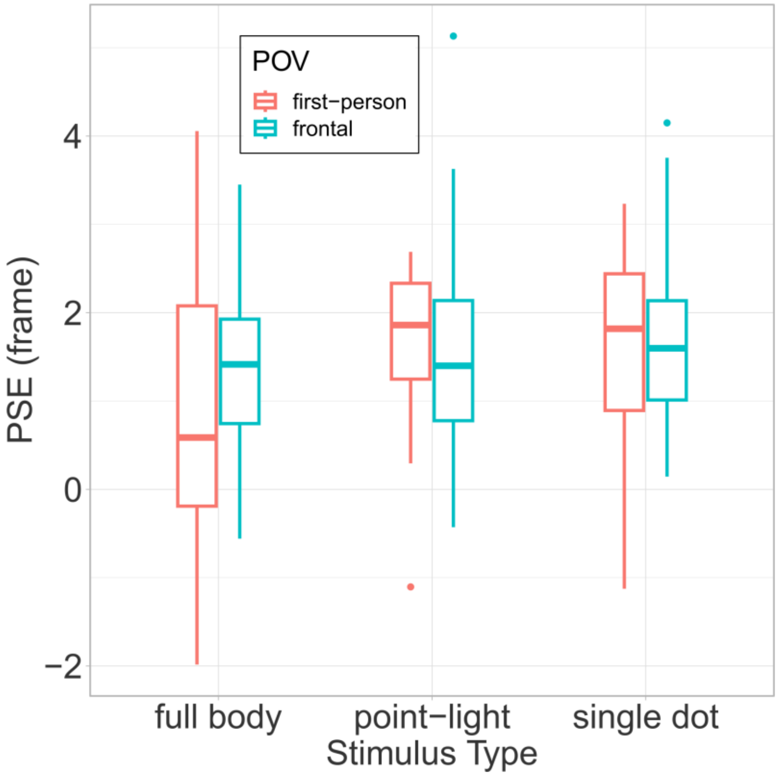

Figure S1 – RM produced in all experimental conditions. The six vertical bars represent the distributions of participants' PSEs when observing human actions from the first-person (red bars) and frontal (cyan bars) point of view, displayed by means of full-body computer-animated avatars (two leftmost bars), point-light stimuli (two central bars) or single-dot stimuli (two rightmost bars). In each bar plot, the inferior and superior borders of the box indicates the 25th (Q1) and 75th (Q3) quantiles of the distribution, the vertical line spans the interval Q1 – 1.5·(Q3-Q1) and Q2 + 1.5·(Q3-Q1), where Q3-Q1 represents the inter-quantile range. Results outside of this interval are plotted as individual dots. Data plotted in this figure are the same as those shown in Figure 4 of the main manuscript, prior to collapsing across the factor point of view.

Individual t-test confirmed that the means of all six distributions were significantly different from 0 (all p-values were Holm-corrected for multiple comparisons): p(full-body, first-person)=0.031, p(full-body, frontal)=$2.3\cdot10^{-5}$, p(point-light, first-person)=$1.7\cdot10^{-6}$, p(point-light, frontal)=$7.5\cdot10^{-5}$, p(single dot, first-person)=$1.2\cdot10^{-5}$, p(single dot, frontal)= $3.1\cdot10^{-6}$.
